# Early bilateral pulmonary embolism in a polytrauma patient: About a case report

**DOI:** 10.1016/j.amsu.2022.103868

**Published:** 2022-05-30

**Authors:** I. Arhoun el haddad, A. El mouhib, O. Hattab, M. Assamti, A. Mojahid, H. Bkiyer, S. Nasri, I. Skiker, El Ouafi, B. Housni

**Affiliations:** a Department of Intensive Care Unit, Mohammed VI University Hospital of Oujda, Mohammed First University of Oujda, Oujda, Morocco; b Department of Cardiology, Mohammed VI University Hospital of Oujda, Mohammed First University of Oujda, Oujda, Morocco; c Department of Radiology, Mohammed VI University Hospital of Oujda, Mohammed First University of Oujda, Oujda, Morocco; d Laboratory of Epidemiology, Clinical Research and Public Health, Faculty of Medicine and Pharmacy, Mohammed the First University of Oujda, Oujda, Morocco

**Keywords:** Venous thromboembolism, Polytrauma, Pulmonary embolism, Anticoagulation therapy, PE, Pulmonary embolism, VTE, Venous thromboembolism, TBI, Traumatic brain injuries, DVT, Deep venous thromboembolism, ICU, Intensive care unit, TAC, Therapeutic anticoagulation

## Abstract

**Introduction:**

and importance: Venous thromboembolism (VTE) is a well-known complication in polytrauma patients, associated with a high rate of mortality and morbidity. Generally pulmonary embolism (PE) is most common between the fifth and seventh days following a significant trauma, and it is uncommon before the fourth day. Their management remains a challenge to physicians given the nature and risk of blood loss from the accompanying injuries must be considered while using anticoagulant therapy.

**Case presentation:**

Here we present a case of acute pulmonary embolism in a previously healthy young woman that developed two days after a traumatic brain injury (TBI) and varying degrees of additional blunt thoracic trauma. An angio CT scan was used to make the diagnosis, and the patient was given anticoagulant medication with close monitoring and satisfactory outcomes.

**Conclusion:**

Evidence suggests that early after trauma, a considerable number of trauma patients are hypercoagulable. In patients with unexplained dyspnea/hypoxia, clinicians should maintain a high index of suspicion and explore PE early after injury. In the case of traumatic brain injury patients with cerebral contusions, intraparenchymal haemorrhages, or subdural/extradural haemorrhages, the existence of post-traumatic PE adds to the problems.

## Highlights

• Our aim was to investigate the (PE) in polytrauma patients: incidence, risk factors of early occurrence, management and outcomes.
• Decision to anti-coagulate post traumatic PE is controversial, especially in polytrauma patients.
• Anticoagulation decision is guided by interval repeat CT scan and clinical picture.
• The presence of post traumatic PE poses further challenges in the management of traumatic brain injury patients with cerebral contusions, intraparenchymal haemorrhages or subdural/extradural haemorrhages.

## 1 Introduction

Traumatic situations are now recognized to raise the risk of VTE, and they are thought to be responsible for about 12% of all VTE episodes in the population. [1], moreover, the PE is the third most common cause of death in injured patient surviving the first 24 hours [2]. Indeed, pulmonary embolism is more common with brain trauma complicated by meningeal bleeding, spine trauma, pelvic trauma, and lower limb trauma, and more rarely thoracic injuries [2]. The occurrence of a pulmonary embolism is often secondary to migration of a thrombus from a DVT, it can occur in the days following a traumatic event, most commonly between the fifth and seventh days, and is uncommon before the fourth day [[4], [5], [6], [7]].

Recent studies suggest that the occurrence of a thromboembolic complication is favored by the combination of three factors: venous stasis, endothelial damage and hypercoagulability (Virchow's triad).In addition, the intense inflammatory reaction associated with polytrauma potentiates the effect of these factors [2].

Given the nature and risk of bleeding from the associated injuries, the decision to start anticoagulant treatment in these patients will be made after consultation with radiologist, cardiologist, neurosurgery, and thoracic surgery, with strict monitoring, and always taking into account the risk-benefit balance.

Our case report was written according to CARE guidelines [3].

## 2 Case presentation

A 40 year old women presented to our emergency department 30 minutes after a 1.5-m stair fall with a cranio-thoracic impact point, although she was alert and oriented at the scene, she had transient amnesia for events immediately surrounding the trauma, there was no history of significant illness, family history was negative for any bleeding disorders, hypertension or diabetes mellitus, and she was on no medications prior to trauma.

On arrival, his vitals were: Glasgow coma scale (GCS) at 14/15, blood pressure was 140/70 mmhg, heart rate 80/min, respiratory rate 30c/min with SaO2 at 84%, capillary glycemia 1.2g/l

**Fig. 1:**
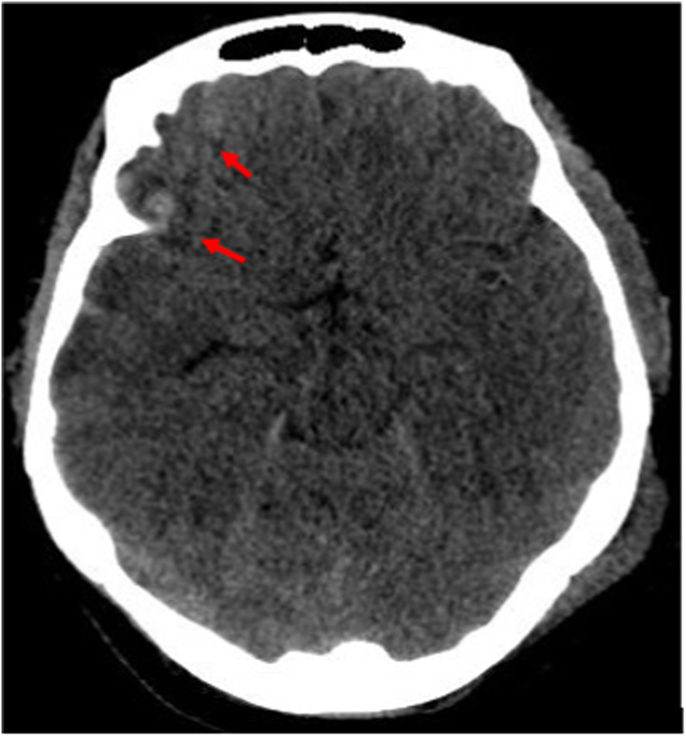

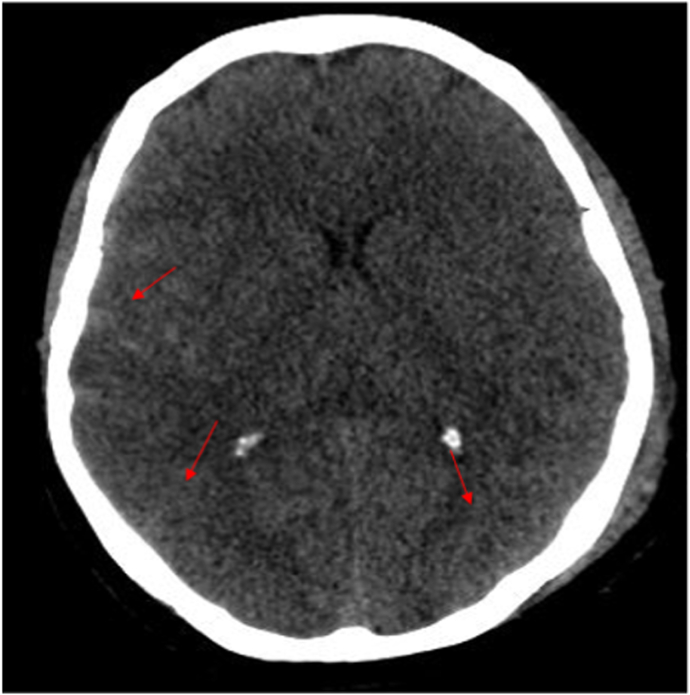
Axial view of a cerebral CT scan revealing A: a moderate meningeal hemorrhage B: hemorrhagic contusion in the parietal, basifrontal, and temporal areas.

She also had a lacerated wound on his left forearm, clotting profile was within normal range. A body scan showed a non-surgical meningeal hemorrhage (Fig. 1,A)with hemorrhagic contusion in the parietal, basifrontal, and temporal areas (Fig. 1,B), a large pneumothorax treated successfully with an intercostal drain, and a fracture of the 5th, 6th and 7th left rib, the patient remained hemodynamically and respiratory stable 1 day later she acutely deteriorated with, tachypnea (RR 35) and hypoxia (SaO_2_ 70% on room air and 91% under oxygen mask), tachycardia at 125bpm, a control X-ray revealed no PNO or effusion, the cardiovascular examination was normal, no lower limb injury and no clinical signs of DVT were identified, no signs or history of documented COVID-19 infection and was fully vaccinated (second dose administered 2 months earlier).

**Fig. 2:**
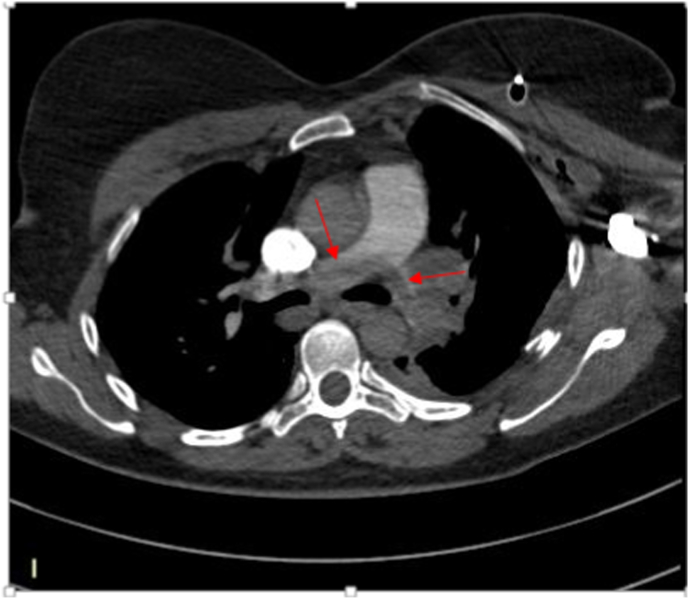

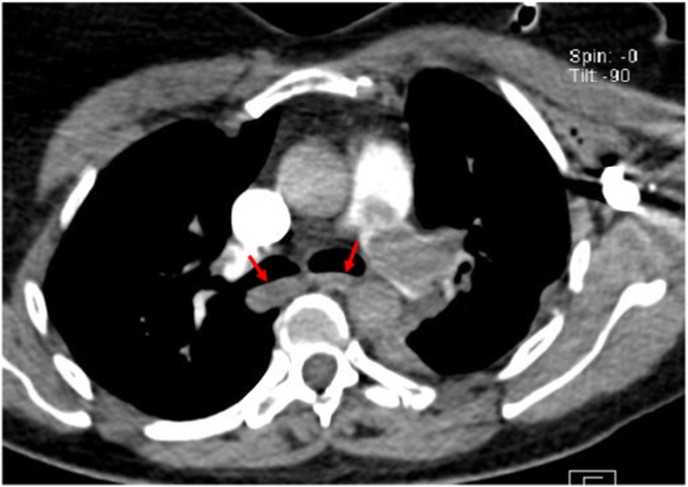
Axial view of a computed tomography pulmonary angiography showing A: pulmonary embolism in the segmental left branch of the left lobe pulmonary artery B: pulmonary embolism in the pulmonary artery trunk.

The patient was transferred to ICU where regular blood tests such as hemogram, coagulation parameters, liver function, and renal function tests were all normal, however the D-dimer level reached 15000 mg/L (the reference limit is 500 mg/L), the diagnosis of pulmonary embolism was suspected and she proceeded to a CT pulmonary angiogram on the same day, objectifying an extensive bilateral proximal pulmonary embolism, deep vein thrombosis was not found on a Doppler ultrasonography of the lower limbs.(Fig. 2:A,B).

A brain scan was performed on day 3 of hospitalization at ICU, indicating a modest reduction of the meningeal hemorrhage, hence, an anticoagulation based on low molecular weight heparin was initiated following agreement of the neurosurgeons.

**Fig. 3:**
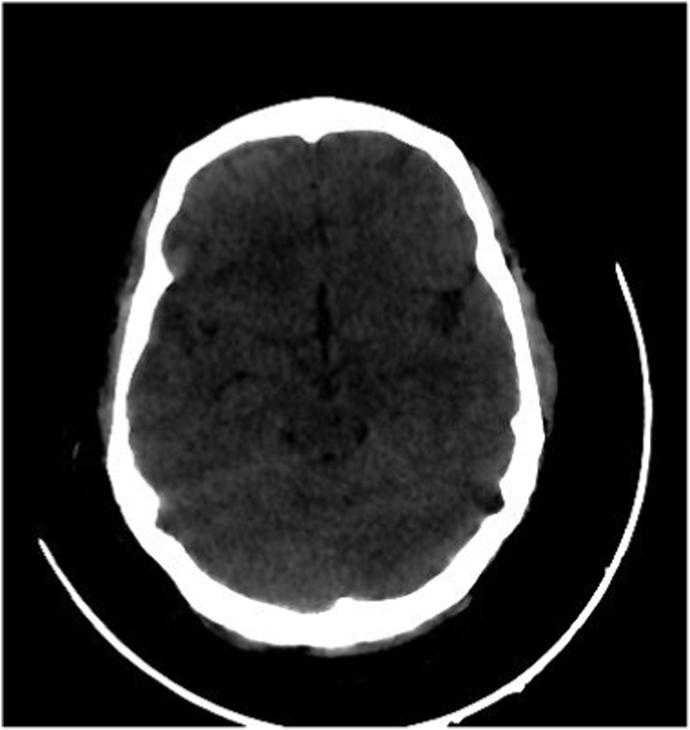
A follow-up brain scan showing a clear regression of the meningeal hemorrhage.

The patient remained stable, she was transferred to intensive care of cardiology, a control scan on day 1 and later on day 12 (Fig. 3) after initiation of heparin therapy, revealed clear meningeal hemorrhage regression, direct oral anticoagulant relay, and respiratory rehabilitation with good clinical evolution.

## 3 Discussion

Post-traumatic thromboembolism is now a common challenging condition, especially among severely ill patients sent to intensive care units, it has been recognized as the third major cause of death among patients who survive the first 24 h following a lesion [2].According to a twenty-month-long prospective cohort study conducted by Bahloul et al. [2] 66 patients of 365 admitted in ICU had a PE post traumatic events,and 27 patients (41.5%) developed the PE within the 72 hours of trauma. In another retrospective study carried out by Dabradi et al. [8], including 240 trauma-patients requiring ICU admission, with a confirmed diagnosis of pulmonary embolism (PE), 48.5% of the patients developed this complication within 72 h following a trauma event and/or after ICU admission.

Generally, dyspnea, tachypnea, chest pain, and hemoptysis are common symptoms. Circulatory instability or shock may also be present in severe situations. The diagnosis is made by, noninvasive methods such as computed tomography pulmonary angiography (CTPA), which shows an obstruction in the pulmonary artery or its branches, and also the echocardiography that can reveal an acute right ventricular dysfunction, dilatation of the pulmonary artery trunk.

Post traumatic PE is a well-known clinical condition that affects up to 24% of people [4,9], though, there was no agreement on the definition "early" or "late" post-traumatic PE, an early PE is defined as one that occurs before the fourth day after a trauma.

Traditionally, the development of VTE has been linked to Virchow's triad including the venous stasis, endothelial vascular damage, and hypercoagulability, leading to EP between the fifth and the seventh day [4,10,11], However, after analyzing the literature, we acknowledges acute inflammation as a fourth factor to the genesis of thrombi as a result of the intensity of the trauma, in fact, it results the production of pro-inflammatory cytokines, which plays a key role in establishing a pro-coagulant state and enhancing endothelial damage. [12,13], this approach could explain why thoracic damage is linked to PE but not to DVT in a significant way.

Early pulmonary embolism in trauma can be explained in two ways, firstly the existence of an undiagnosed thrombophilia, either congenital or acquired after trauma, [14]. Velmahos [15] proposed the second theory, suggesting that the majority of patients with early post-traumatic PE were not identified with DVT based on computed tomographic venography of the pelvis and lower leg veins, implying that pulmonary clots can form "de novo" within the lungs.

Several risk factors of occurrence of PE were established including elder age, obesity, long bone fractures, hypoxemia,sepsis and severe injury [7,10,11],more recently higher injury severity score (ISS) and the existence of long-bone fractures in the lower extremities were discovered by Darabadi [8] as two independent factors of early PE incidence, our patient present a multivariate risk factors of PE including the brain bleeding, pneumothorax drainage with a minor ISS,however the early occurrence seems to be more likely due to “in situ” formation of a clot in the peripheral pulmonary arteries caused by direct inflammation of pulmonary vessels after injury.

Decision to anti-coagulate post traumatic PE is controversial, given its risk of increased intracranial hemorrhage expansion, and poses further challenges in the management of traumatic brain injury patients with cerebral contusions, intraparenchymal haemorrhages or subdural/extradural haemorrhages, therefore it was insufficiently discussed in the literature.

In a retrospective single center study conducted by Amanda M.chipman [16], early TAC was defined as within 7 days of injury or less; late was defined as after 7 days, among 46 patients, 19 received early TAC and 27 received late TAC, traumatic intracranial hemorrhage occurred in three patients of the early group and two of the late, in addition, this study shows that therapeutic AC, even if started within 7 days of injury, is not associated with worse results in TBI patients. These findings imply that, while starting therapeutic AC for ICH patients should be done with caution, it is generally safe to do so with appropriate monitoring and follow-up imaging, even early after injury.

In another retrospective study carried out by Kazuhide Matsushima et al. [17], among 3351 TBI patients, 72 received TAC, the most prevalent post-injury indication was venous thromboembolism, TAC was started 9 days after the injury on average, the intravenous heparin infusion was the most commonly used agent for TAC (70.8%),In six patients, a repeat head CT revealed progression of hemorrhagic TBI without any signs of neurologic deterioration, the age of 65 years was found to be a significant determinant in the progression of hemorrhagic TBI after TAC initiation.

Reviewing studies, we found that the chemical thromboprophylaxis based on low-dose subcutaneous low molecular weight (40 mg/day) or unfractionated heparin (2500–5000 UI/12h) significantly reduces the incidence of VTE in TBI patients with a stable or improved head CT after 24 hours and does not enhance the risk of intracranial hemorrhage development [[18], [19], [20]].

In our case, an early TAC was initiated at the fourth day, initially with LHPM,the patient undergone repeat CT scan control,without intracranial hemorrhage progression, she was discharged on the thirteen day after TAC initiation under new oral direct anticoagulation with good clinical outcomes.

## 4 Conclusion

PE (post-traumatic pulmonary embolism) is the third most prevalent cause of death in trauma patients and remains a serious problem in cardio-pulmonary disorders. Early occurrence is still uncommon and associated with a poor prognosis, patient with PE and concomitant traumatic brain injury have competing healthcare requirements that necessitate a careful balance of anticoagulant versus potential worsening of their injuries.

## Provenance and peer review

Not commissioned, externally peer-reviewed.

## Sources of funding for your research

None.

## Ethical approval

The ethical committee approval was not required give the article type (case report).However, the written consent to publish the clinical data of the patients was given and is available to check by the handling editor if needed.

## Consent

Obtained.

## Author contribution

Inass Arhoun El Haddad^:^ Study concept, Data collection, Data analysis, Literature research, writing the paper. Amine El Mouhib: Data collection, Data analysis Oumayma Hattab: Study concept, Data collection, Data analysis, Literature research, writing the paper Maryem Assamti: Data collection, Data analysis Amal Mojahid: Data collection, Data analysis Houssam Bkiyer:Supervision and data validation. Siham Nasri: Supervision and data validation. Imane Skiker: Supervision and data validation. Noha El ouafi: Supervision and data validation.Brahim Housni: Supervision and data validation.

## Registration of research studies

This is not an original research project involving human participants in an interventional or an observational study but a case report. This registration is was not required.

## Guarantor

Inas Arhoun el Haddad/Oumayma Hattab.

## Conflicts of interest

None.
